# Guest Editorial: Papers from the 13th Workshop on Augmented Environments for Computer Assisted Interventions

**DOI:** 10.1049/htl.2019.0102

**Published:** 2019-12-02

Welcome to this Special Issue of the IET's *Healthcare Technology Letters* (HTL) journal dedicated to the joint edition of the 8th Medical Imaging and Augmented Reality (MIAR), the Augmented Environments for Computer-Assisted Interventions (AE-CAI), and the 6th Computer Assisted and Robotic Endoscopy (CARE) workshops. We are pleased to present the proceedings of this exciting scientific gathering held in conjunction with the Medical Image Computing and Computer-Assisted Interventions (MICCAI) conference on October 13th, 2019 at in Shenzhen, China.

Over the past several years, the satellite workshops and tutorials at MICCAI have experienced increased popularity. This year's workshop brings together three communities that joined forces back in February 2019 in light of our common interests in image guidance, navigation and visualization for computer-assisted interventions. MIAR / AE-CAI / CARE 2019 is a joint venture between the prestigious Medical Imaging and Augmented Reality (MIAR) workshop series founded in 2002, the series of MICCAI-affiliated workshops on Augmented Environments for Computer-Assisted Interventions (AE-CAI) founded in 2006 and featured annually ever since, and the Computer Assisted and Robotic Endoscopy (CARE) workshop series, now on its 6th edition. This year's edition of the workshop received 30 submissions and reached over 60 registrants, not including the members of the organizing and program committees, making MIAR/AE-CAI/CARE one of the best received and best attended workshops with more than a decade-long standing tradition at MICCAI 2019.

Computer-Assisted Interventions (CAI) is a field of research and practice, where medical interventions are supported by computer-based tools and methodologies. CAI systems enable more precise, safer, and less invasive interventional treatments by providing enhanced planning, real-time visualization, instrument guidance and navigation, as well as situation awareness and cognition. These research domains have been motivated by the development of medical imaging and its evolution from being primarily a diagnostic modality towards its use as a therapeutic and interventional aid, driven by the need to streamline the diagnostic and therapeutic processes via minimally invasive visualization and therapy. To promote this field of research, our workshop seeks to showcase papers that disseminate novel theoretical algorithms, technical implementations, and development and validation of integrated hardware and software systems in the context of their dedicated clinical applications.

The objective of the joint workshop has been to attract scientific contributions that offer solutions to the technical problems in the area of augmented and virtual environments for computer-assisted and robotic interventions to provide a venue for dissemination of papers describing both complete systems and clinical applications. The workshop attracts researchers in computer science, biomedical engineering, computer vision, robotics, and medical imaging.

The workshop featured a single track that consisted of long and short oral presentations showcasing original research engaged in the development of virtual and augmented environments for medical image visualization and image-guided interventions. To foster networking and discussion, we also provided all authors with the opportunity to present their work as a poster in addition to their oral presentation slot.

All manuscripts submitted to the joint MIAR / AE-CAI / CARE 2019 were held up to journal standards, as the ultimate objective was to publish accepted work in this Special Issue of the IET's *Healthcare Technology Letters* journal. This year's joint workshop received 30 manuscripts spanning a strong geographic representation from Europe, North America and Asia. The review process was rigorous and involved evaluation of each manuscript by three to five external reviewers. Following the first stage of review, sixteen manuscripts were further considered for major or minor revision, and resubmission. Authors were required to submit a response to reviewers and resubmit a revised manuscript that addressed all reviewers’ critiques.

Once revised, all resubmitted manuscripts entered a second round of review conducted by the Program Committee, Workshop Chairs, as well as the HTL Managing Editor and Editor-in-Chief to ensure that all reviewers’ critiques were properly addressed in the revised manuscripts and that the quality of the manuscript was appropriate for journal publication. All manuscript were then accepted for publication and forwarded to the journal for production and publication.

In addition to the contributed papers, we were pleased to welcome Dr. Kensaku Mori – Professor in the Graduate School of Informatics and Director of the Information Technology Center at Nagoya University – who spoke on the development and use of artificial intelligence for endoscopic procedures.

On behalf of the MIAR, AE-CAI and CARE 2019 Program and Organizing Committee, we would like to extend our sincere gratitude to all authors, presenters, and attendees for their scientific contribution, enthusiasm, and support. We also extend special thanks to all reviewers for providing detailed and timely critiques of the submitted manuscripts. We also acknowledge the support we received from the MICCAI 2019 Workshop Committee, and the HTL Editorial Office for their dedication to maintaining the high quality of the workshops, and for selecting outstanding manuscripts that fostered exciting discussions at the meeting.

Last but not least, we acknowledge our generous sponsors. We thank *Northern Digital Inc. (NDI)* for their continued support over the years, and we also welcome and thank *Intuitive Surgical* as a new sponsor for our workshop. Our sponsors’ generous contributions have enabled us to recognize our authors for their much deserved dedication and scientific enthusiasm through several paper awards. A big thank you on behalf of all awardees and once again, sincere congratulations to all award winners! The award-winning papers appearing in this Special Issue are listed below!

- Andinet Enquobahrie *et al*. Development and Face Validation of Ultrasound-Guided Renal Biopsy Virtual Trainer
- Megha Kalia *et al*. A Marker-less Real Time Intra-Operative Camera and Hand-Eye Calibration Procedure for Surgical Augmented Reality
- Reid Vassallo *et al*. Determining blood flow direction from short neurovascular surgical microscope videos
- Sara M. S. Amini *et al*. An Augmented Reality Mastectomy Surgical Planning Prototype using the HoloLens
- Mohammad Ali Armin *et al*. Learning colon centerline from optical colonoscopy, a new way to generate a map of the internal colon surface
- Leah Groves *et al*. A deep learning approach for automatic out-of-plane needle localization for semiautomatic ultrasound probe calibration

We hope that you will enjoy reading this Special Issue and we look forward to your continuing support and participation in future editions of the MIAR, AE-CAI & CARE workshops. Their continued success demands our ongoing commitment.

## Guest Editors:

Cristian A. Linte, Rochester Institute of Technology, United States

Xiongbiao Luo, Xiamen Medical University, China

Jonathan McLeod, Intuitive Surgical, United States

Ziv Yaniv, National Institutes of Health and TAJ Technologies, United States

## Guest Associate Editors:

Elvis C. S. Chen, Western University, Canada

Marta Kersten-Oertel, Concordia University, Canada

Hongen Liao, Tsinghua University, China

Yuan Zhang, University of Jinan

